# Clinical Characteristics and Safety Profiles of Japanese Psoriasis Patients Who Continued Apremilast Treatment for 6 and 12 Months: A Post Hoc Analysis of an Apremilast Postmarketing Surveillance Study

**DOI:** 10.1111/1346-8138.17764

**Published:** 2025-05-10

**Authors:** Mamitaro Ohtsuki, Yukari Okubo, Hidehisa Saeki, Atsuyuki Igarashi, Shinichi Imafuku, Masatoshi Abe, Katsuya Saito, Ryuichi Ogawa, Akimichi Morita

**Affiliations:** ^1^ Department of Dermatology Jichi Medical University Shimotsuke Japan; ^2^ Department of Dermatology Tokyo Medical University Tokyo Japan; ^3^ Department of Dermatology Nippon Medical School Tokyo Japan; ^4^ Igarashi Dermatology Higashi‐Gotanda Clinic Tokyo Japan; ^5^ Department of Dermatology Fukuoka University Fukuoka Japan; ^6^ Kojinkai Sapporo Skin Clinic Sapporo Japan; ^7^ Medical Affairs, Amgen K.K. Tokyo Japan; ^8^ Department of Geriatric and Environmental Dermatology Nagoya City University Graduate School of Medical Sciences Aichi Japan

**Keywords:** apremilast, Japan, postmarketing product surveillance, psoriasis, safety

## Abstract

Apremilast is a phosphodiesterase 4 inhibitor approved for moderate to severe psoriasis in Japan. Apremilast significantly improved Physician's Global Assessment (PGA) and Dermatology Life Quality Index (DLQI) both at 6 and 12 months in a previously published primary post‐surveillance study. Here, we performed a post hoc analysis of the surveillance data to evaluate patient characteristics, effectiveness, and safety among psoriasis patients who continued apremilast for 6 and 12 months. The PMS included 992 patients, of whom 646 of 992 patients continued treatment for 6 months and 509 of 992 patients subsequently continued treatment for 12 months. Baseline characteristics between these groups were similar. Among 992 patients, the treatment persistence rate was 65.1% at 6 months and 51.3% at 12 months after the start of apremilast treatment. PGA 0/1 response was 47.9% at 6 months and 60.8% at 12 months, whereas DLQI 0/1 responses at 6 months and 12 months were 38.5% and 58.7%, respectively. Among 646 patients who continued apremilast for 6 months, diarrhea was reported in 60 patients (9.3%), nausea in 35 patients (5.4%), and headache in 11 (1.7%) patients, which were mainly observed within the first month since treatment initiation. In 509 patients who continued apremilast for 12 months, diarrhea was reported in 43 patients (8.5%), nausea in 24 patients (4.7%), and headache in 6 (1.2%) patients; similar frequencies of these adverse reactions were observed within 6 months and between 6 and 12 months of follow‐up. It is important to continue apremilast by appropriately managing diarrhea and nausea in real‐world practice.

## 1 Introduction

Psoriasis, a chronic, inflammatory, multisystem disease, not only affects the skin but also impacts comorbid burden potentially resulting in psoriatic arthritis and metabolic syndrome/dysfunction. It is an immune‐mediated disease characterized by excessive stimulation of T cells, leading to the formation of thickened, scaly plaques on the skin [1].

Apremilast (Otezla tablets, Amgen K.K., Tokyo, Japan), a phosphodiesterase 4 inhibitor, suppresses the production of cytokines involved in psoriasis pathogenesis [2]. Apremilast efficacy and safety have been confirmed in international clinical trials [3, 4, 5, 6, 7], but real‐world clinical practice data in Japanese psoriasis patients are limited. Recently, a postmarketing surveillance study of apremilast we published showed that orally administered apremilast was well tolerated and effective in Japanese patients with plaque psoriasis (PsO) and/or psoriatic arthritis (PsA), with a consistent safety profile in real‐world settings relative to randomized clinical trials with no new safety signals [8]. Here, we performed a post hoc analysis of the postmarketing surveillance study and evaluated the patient characteristics, persistence rate, effectiveness, and safety profiles of psoriasis patients who continued apremilast during the first 6 months and between 6 and 12 months.

## 2 Methods

Detailed methods are described in the previous postmarketing surveillance study paper [8]. Briefly, this multicenter, prospective, observational study (ClinicalTrials.gov identifier: NCT03284879; European Union electronic Register of Post‐Authorisation Safety Studies [EU PAS] identifier: EUPAS36684) was conducted in Japan in patients with plaque psoriasis and/or psoriatic arthritis treated with apremilast in routine clinical practice. Eligible patients had received apremilast for the first time for plaque psoriasis with an inadequate response to topical therapies and/or for psoriatic arthritis. Excluded patients had a history of hypersensitivity to apremilast or to any formulation excipient(s). Patients initially received apremilast at a dose of 10, 20, or 30 mg, in accordance with the Japanese prescribing information [2]. A total of 1086 patients were enrolled at 160 hospitals or clinics in Japan between September 1, 2017, and August 31, 2019, and were observed for 12 months after the start of apremilast or until discontinuation or withdrawal in the original postmarketing surveillance study. Six‐month patient continuations were acknowledged upon completion of Survey 1 (6 months after start of treatment), whereas 12‐month patient continuations were acknowledged upon completion of Survey 2 (6 months to 1 year after start of treatment). This post hoc analysis focused on 992 plaque psoriasis patients assessable for the safety of apremilast based on having collected data and not being excluded for late enrollment, not receiving treatment, or not returning to the hospital after treatment initiation. Patients with psoriatic arthritis were excluded as the focus was on PsO patients and patients who continued taking apremilast for 6 and 12 months as the analysis target population. Treatment effectiveness was evaluated by Physician's Global Assessment (PGA) scores of 0/1 and Dermatology Life Quality Index (DLQI) scores of 0/1. Adverse events were classified using the Japanese version of the Medical Dictionary for Regulatory Activities (MedDRA/J) version 25.0. Adverse events reported by physicians other than those assessed as “not related” by physicians (including unknown/not specified) were tabulated as adverse reactions. Data were summarized using descriptive statistics with the number and proportion of patients. Loss to follow up was not included in the analysis because the information required for the analysis is unavailable. No statistical testing was performed in this post hoc analysis.

## 3 Results

### 3.1 Baseline Characteristics and Treatment Patterns

The safety analysis set consisted of 992 psoriasis patients, of whom 646 patients continued apremilast for 6 months and 509 for 12 months. In the overall population, most patients were male (70.4%), age ≥ 15 to < 65 years (58.3%), previously treated (86.9%), and categorized as having moderate psoriasis (50.6%). Baseline characteristics (e.g., age, sex, psoriasis severity, comorbidities) between 6‐ and 12‐month groups were similar (Table 1). Although not a focus of this study, the discontinuation group showed no significant differences in patient background to that of the continuation group (data not shown).

**TABLE 1:** Baseline characteristics and treatment patterns of psoriasis patients treated with apremilast.

	Overall PsO patients *N* = 992	Patients with 6 months follow‐up *N* = 646	Patients with 12 months follow‐up *N* = 509
Sex, *n* (%)			
Male	698 (70.4)	453 (70.1)	364 (71.5)
Female	294 (29.6)	193 (29.9)	145 (28.5)
Age category, *n* (%)			
15 years	1 (0.1)	1 (0.2)	1 (0.2)
≥ 15 to < 65 years	578 (58.3)	384 (59.4)	299 (58.7)
≥ 65 years	412 (41.5)	260 (40.2)	208 (40.9)
Unknown	1 (0.1)	1 (0.2)	1 (0.2)
Pretreatment severity (PGA score), *n* (%)			
1. Minor	23 (2.3)	15 (2.3)	11 (2.2)
2. Mild	136 (13.7)	93 (14.4)	77 (15.1)
3. Moderate	502 (50.6)	324 (50.2)	256 (50.3)
4. High	177 (17.8)	118 (18.3)	94 (18.5)
5. Extremely high	27 (2.7)	16 (2.5)	12 (2.4)
Disease duration, *n* (%)			
< 1 year	155 (15.6)	97 (15.0)	66 (13.0)
≥ 1 to < 2 years	68 (6.9)	48 (7.4)	43 (8.4)
≥ 2 to < 5 years	137 (13.8)	89 (13.8)	63 (12.4)
≥ 5 years	480 (48.4)	305 (47.2)	251 (49.3)
Unknown	152 (15.3)	107 (16.6)	86 (16.9)
Alcohol, *n* (%)	298 (30.0)	205 (31.7)	155 (30.5)
Smoking, *n* (%)	263 (26.5)	168 (26.0)	132 (25.9)
History of allergy, *n* (%)	64 (6.5)	45 (7.0)	29 (5.7)
Hospitalization, *n* (%)			
Inpatient	10 (1.0)	7 (1.1)	4 (0.8)
Outpatient	982 (99.0)	639 (98.9)	505 (99.2)
eGFR category, *n* (%)			
≥ 90 mL/min/1.73 m^2^	400 (40.3)	275 (42.6)	219 (43.0)
≥ 60 to < 89 mL/min/1.73 m^2^	256 (25.8)	163 (25.2)	130 (25.5)
≥ 45 to < 59 mL/min/1.73 m^2^	66 (6.7)	40 (6.2)	29 (5.7)
≥ 30 to < 44 mL/min/1.73 m^2^	16 (1.6)	11 (1.7)	9 (1.8)
≥ 15 to < 30 mL/min/1.73 m^2^	1 (0.1)	1 (0.2)	1 (0.2)
< 15 mL/min/1.73 m^2^	4 (0.4)	4 (0.6)	4 (0.8)
Past medical history^a^	235 (23.7)	154 (23.8)	120 (23.6)
Liver disorder	12 (1.2)	8 (1.2)	7 (1.4)
Kidney disorder	10 (1.0)	4 (0.6)	3 (0.6)
Psychiatric disorder	10 (1.0)	3 (0.5)	2 (0.4)
Infection	29 (2.9)	20 (3.1)	18 (3.5)
Comorbidities^b^	494 (49.8)	335 (51.9)	259 (50.9)
Liver disorder	48 (4.8)	41 (6.3)	36 (7.1)
Kidney disorder	66 (6.7)	40 (6.2)	30 (5.9)
Psychiatric disorder	20 (2.0)	13 (2.0)	8 (1.6)
Infection	35 (3.5)	25 (3.9)	17 (3.3)
Prior therapy			
Prior use of medications, *n* (%)	862 (86.9)	571 (88.4)	453 (89.0)
Prior use of phototherapy, *n* (%)	194 (19.6)	135 (20.9)	113 (22.2)
Targeted therapy	36 (3.6)	28 (4.3)	21 (4.1)
Narrow‐band UVB therapy	162 (16.3)	111 (17.2)	97 (19.1)
Concomitant medications, *n* (%)	893 (90.0)	587 (90.9)	464 (91.2)
Concomitant therapy for psoriasis, *n* (%)	857 (86.4)	568 (87.9)	448 (88.0)
Immunosuppressive drugs (cyclosporine, methotrexate)	25 (2.5)	20 (3.1)	16 (3.1)
Vitamin A derivatives (retinoid)	9 (0.9)	7 (1.1)	6 (1.2)
Biologics	7 (0.7)	4 (0.6)	3 (0.6)
Topical corticosteroids	780 (78.6)	520 (80.5)	417 (81.9)
Topical vitamin D3	703 (70.9)	478 (74.0)	383 (75.2)
Phototherapy	209 (21.1)	146 (22.6)	122 (24.0)
Targeted therapy	54 (5.4)	40 (6.2)	29 (5.7)
Narrow‐band UVB therapy	167 (16.8)	117 (18.1)	103 (20.2)
Other	198 (20.0)	140 (21.7)	107 (21.0)

Abbreviations: eGFR, estimated glomerular filtration rate; PGA, Physician Global Assessment; PsO, plaque psoriasis; UV, ultraviolet.

^a^ Disease or symptom that was cured before apremilast initiation.

^b^ Disease or symptom present at apremilast initiation.

In 992 patients with psoriasis, the treatment persistence rate of apremilast was 65.1% (646 of 992 patients) at 6 months and 51.3% (509 of 992 patients) at 12 months after the start of treatment. Further, 78.8% (509 of 646 patients) continued apremilast from month 6 to 12.

### 3.2 Effectiveness of Apremilast in Patients With Psoriasis

**FIGURE 1:**
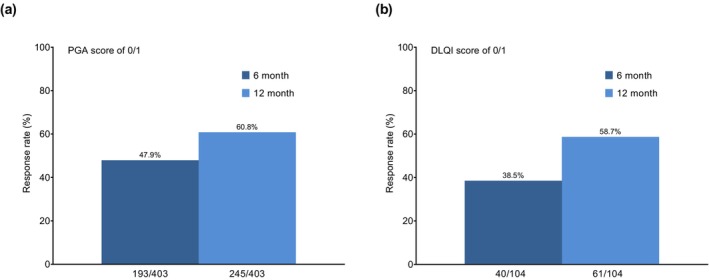
Response rate for (a) Physician's Global Assessment (PGA) 0/1 and (b) Dermatology Life Quality Index (DLQI) in patients with plaque psoriasis.

Analysis of the effectiveness at 6 months and 12 months in patients who continued apremilast for 12 months showed that PGA 0/1 responses were 47.9% (193 of 403 patients) and 60.8% (245 out of 403 patients), respectively (Figure 1a). DLQI 0/1 responses at 6 months and 12 months were 38.5% (40 of 104 patients) and 58.7% (61 of 104 patients), respectively (Figure 1b).

### 3.3 Adverse Reactions in Psoriasis Patients Who Continued Apremilast Treatment

Among the 646 psoriasis patients (65.1%) who continued apremilast for 6 months, 151 patients (23.4%) experienced adverse reactions (Table 2). Diarrhea was observed in 60 patients (9.3%), nausea in 35 patients (5.4%), and headache in 11 patients (1.7%). Most patients took no action or reduced their dose; over 90% (97 of 106 patients) reached “recovered” or “recovering” status, and 43 patients of those reached “recovered” or “recovering” status within 1 month. Actions regarding apremilast in these 43 patients included no action (*n* = 26), dose reduction (*n* = 14), interruption (*n* = 2), and discontinuation (*n* = 1) but it was not subsequently confirmed whether recovery was affected by these actions. Of 509 patients who continued apremilast for 12 months, diarrhea was reported in 43 patients (8.5%) and nausea in 24 patients (4.7%). There was no increase in the frequencies of those or other new adverse reactions observed between 6 and 12 months of follow‐up.

**TABLE 2:** Observed adverse reactions in patients who continued apremilast.

Type of adverse reaction, *n* (%)	Patients with 6 months follow‐up *N* = 646	Patients with 12 months follow‐up *N* = 509
All types, at least one adverse reaction	151 (23.4)	104 (20.4)
Infections and infestations	2 (0.3)	2 (0.4)
Body tinea	1 (0.2)	1 (0.2)
Tonsillitis	1 (0.2)	1 (0.2)
Neoplasms benign, malignant and unspecified (including cysts and polyps)	1 (0.2)	1 (0.2)
Hepatocellular carcinoma	1 (0.2)	1 (0.2)
Metabolism and nutrition disorders	6 (0.9)	5 (1.0)
Decreased appetite	6 (0.9)	5 (1.0)
Psychiatric disorders	3 (0.5)	2 (0.4)
Alcoholic hangover	1 (0.2)	1 (0.2)
Depression	1 (0.2)	1 (0.2)
Depressive symptom	1 (0.2)	0 (0.0)
Nervous system disorders	17 (2.6)	9 (1.8)
Headache	11 (1.7)	6 (1.2)
Dizziness	3 (0.5)	2 (0.4)
Cerebral infarction	1 (0.2)	1 (0.2)
Dysgeusia	1 (0.2)	0 (0.0)
Taste disorder	1 (0.2)	0 (0.0)
Ear and labyrinth disorders	1 (0.2)	1 (0.2)
Vertigo	1 (0.2)	1 (0.2)
Cardiac disorders	1 (0.2)	1 (0.2)
Myocardial infarction	1 (0.2)	1 (0.2)
Respiratory, thoracic, and mediastinal disorders	1 (0.2)	0 (0.0)
Laryngeal discomfort	1 (0.2)	0 (0.0)
Gastrointestinal disorders	120 (18.6)	86 (16.9)
Diarrhea	60 (9.3)	43 (8.5)
Nausea	35 (5.4)	24 (4.7)
Feces soft	25 (3.9)	18 (3.5)
Abdominal discomfort	9 (1.4)	7 (1.4)
Dyspepsia	5 (0.8)	4 (0.8)
Abdominal pain	2 (0.3)	2 (0.4)
Vomiting	4 (0.6)	2 (0.4)
Frequent bowel movements	2 (0.3)	2 (0.4)
Gastritis	1 (0.2)	0 (0.0)
Gastroesophageal reflux disease	1 (0.2)	1 (0.2)
Skin and subcutaneous tissue disorders	8 (1.2)	4 (0.8)
Psoriasis	5 (0.8)	3 (0.6)
Erythema	1 (0.2)	1 (0.2)
Drug eruption	1 (0.2)	0 (0.0)
Rash	1 (0.2)	0 (0.0)
Musculoskeletal and connective tissue disorders	1 (0.2)	1 (0.2)
Psoriatic arthropathy	1 (0.2)	1 (0.2)
General disorders and administration site conditions	5 (0.8)	3 (0.6)
Therapeutic response decreased	2 (0.3)	0 (0.0)
Feeling abnormal	1 (0.2)	1 (0.2)
Malaise	1 (0.2)	1 (0.2)
Oedema peripheral	1 (0.2)	1 (0.2)
Investigations	4 (0.6)	3 (0.6)
Alanine aminotransferase increased	1 (0.2)	1 (0.2)
Glycosylated hemoglobin increased	1 (0.2)	0 (0.0)
Lymphocyte count decreased	1 (0.2)	1 (0.2)
Weight decreased	1 (0.2)	1 (0.2)

## 4 Discussion

This post hoc analysis evaluated the effectiveness and occurrence of adverse reactions using a real‐world data set comprising approximately 1000 Japanese psoriasis patients who continued apremilast treatment. The real‐world nature of the data distinguishes this study from previous clinical studies and also enhances its clinical applicability. Baseline characteristics between 6‐ and 12‐month groups were similar and persistence at 6 months and 12 months after the start of treatment were 65.1% and 51.3%, respectively. We have previously reported from this postmarketing surveillance study that patients who continued apremilast treatment showed significant improvements in clinical evaluations such as PGA and patient‐reported outcomes such as DLQI both at 6 and 12 months of treatment [8], indicating that continued apremilast treatment can improve quality of life and relieve disease burden associated with psoriasis. The most common adverse reactions during apremilast treatment were gastrointestinal disorders, especially diarrhea and nausea. However, as with adverse reactions overall, gastrointestinal adverse reactions generally resolved within a month of onset of observation.

To enable persistent apremilast treatment in plaque psoriasis patients, it is important to manage gastrointestinal disorders during apremilast treatment, especially in the first 3–6 months after treatment initiation. In 2021, Daudén Tello et al. recommended multidisciplinary management of apremilast effects [9]. The adverse effects caused by apremilast therapy are usually self‐limiting and resolve spontaneously in the initial phases (1 month) of treatment; they recommend anti‐secretory agents (e.g., racecadotril or loperamide) for diarrhea and antiemetics (e.g., ondansetron or metoclopramide) for nausea if pharmacological treatment is required [9]. We acknowledge a lack of statistical testing related to efficacy endpoints as a limitation of this study because it was not included as part of the original statistical analysis plan.

## 5 Conclusion

Japanese real‐world data uncovered that Japanese psoriasis patients generally continued apremilast and received benefits from long‐term systemic treatment despite experiencing adverse reactions. Adequate management of gastrointestinal symptoms in the early stage of treatment and continued persistence with apremilast are expected to improve patients' quality of life and provide long‐term disease control.

## Conflicts of Interest

Mamitaro Ohtsuki has received support for the present manuscript from Amgen K.K.; grants or contracts from AbbVie, Boehringer Ingelheim, Mitsubishi Tanabe Pharma, Maruho Co. Ltd., Sun Pharma Japan, and Taiho Pharmaceutical Co. Ltd.; consulting fees from Amgen K.K.; payment or honoraria for lectures, presentations, speakers' bureaus, manuscript writing or educational events from AbbVie, Amgen K.K., Bristol Myers Squibb, Janssen Pharmaceutical, Lilly, Maruho Co. Ltd., and UCB; participation on a Data Safety Monitoring Board or Advisory Board from Boehringer Ingelheim, Bristol Myers Squibb, and UCB. Akimichi Morita has received research grants, consultancy fees, and/or speaker's fees from AbbVie, Amgen, Boehringer‐Ingelheim, Bristol‐Myers Squibb, Eisai, Eli Lilly Japan, Janssen Pharmaceutical, Kyowa Kirin, LEO Pharma, Maruho Co. Ltd., Minophagen Pharmaceutical, Mitsubishi Tanabe Pharma, Nippon Kayaku, Novartis, Pfizer Japan, Sun Pharma Japan, Taiho Pharmaceutical, Torii Pharmaceutical, UCB Japan, and Ushio. Yukari Okubo has received grants, consultancy fees, and/or speaker's fees from Sun Pharma Japan, Maruho Co. Ltd., Pfizer Japan, AbbVie, Amgen, Boehringer Ingelheim, Bristol Myers Squibb, Eli Lily and Company, Janssen Pharma, Kyowa Kirin, LEO Pharma, Mitsubishi Tanabe, Otsuka Pharmaceutical, Sanofi, Taiho, Torii, and UCB Pharma. Hidehisa Saeki has received research grants, consultancy fees, and/or speaker's fees from Amgen, Mitsubishi Tanabe Pharma, Taiho Pharmaceutica, Torii Pharmaceutical, Maruho Co. Ltd., AbbVie, Novartis Pharma, Eli Lilly Japan, Eisai, LEO Pharma, UCB Japan, Bristol Myers Squibb, Nippon Boehringer Ingelheim, and Sun Pharma Japan. Atsuyuki Igarashi has received consultancy fees and/or speaker's fees from AbbVie Inc., Amgen K.K., Eli Lilly Japan K.K., Janssen Pharmaceutical K.K., Kyowa Kirin Co., LEO Pharma Ltd., Novartis Pharma K.K., Maruho Co. Ltd., Sun Pharma Japan Ltd., Taiho Pharmaceutical Co. Ltd., Torii Pharmaceutical Co. Ltd., Japan Tobacco Inc., and UCB Japan. Shinichi Imafuku has received grants and/or personal fees from AbbVie, Amgen, Behringer Ingelheim, Bristol Myers Squibb, Eisai, GSK, Janssen, Kyowa Kirin, LEO, Lilly, Maruho Co. Ltd., Novartis, Pfizer, Sankyo‐Daiichi, Sun Pharma Japan, Taiho Yakuhin, Torii Yakuhin, and UCB. Masatoshi Abe has received research grants, consulting fees, speaker fees, and/or participated in clinical trials for AbbVie, Amgen, Bristol Myers Squibb, Eli Lilly, Kyowa Kirin, LEO Pharma, Maruho Co. Ltd., Novartis, Otsuka, Sun Pharma Japan, Sanofi, Torii, and UCB. Katsuya Saito and Ryuichi Ogawa are employees of Amgen K.K. Hidehisa Saeki and Shinichi Imafuku are Editorial Board members of *Journal of Dermatology* and co‐authors of this article. To minimize bias, they were excluded from all editorial decision‐making related to the acceptance of this article for publication.

## Funding Statement

Amgen K.K. conducted this study based on instructions from the Pharmaceutical and Medical Devices Agency, therefore, no funds or grants were received.

## Data Availability

The datasets used and/or analyzed during the current study are not available.
